# Neuronal primary cilia integrate peripheral signals with metabolic drives

**DOI:** 10.3389/fphys.2023.1150232

**Published:** 2023-03-29

**Authors:** Kelly M. DeMars, Madeleine R. Ross, Alana Starr, Jeremy C. McIntyre

**Affiliations:** ^1^ Department of Neuroscience, University of Florida, Gainesville, FL, United States; ^2^ Summer Neuroscience Internship Program, University of Florida, Gainesville, FL, United States

**Keywords:** primary cilium, hypothalamus, metabolism, obesity, BBSome, intraflagellar transport

## Abstract

Neuronal primary cilia have recently emerged as important contributors to the central regulation of energy homeostasis. As non-motile, microtubule-based organelles, primary cilia serve as signaling antennae for metabolic status. The impairment of ciliary structure or function can produce ciliopathies for which obesity is a hallmark phenotype and global ablation of cilia induces non-syndromic adiposity in mouse models. This organelle is not only a hub for metabolic signaling, but also for catecholamine neuromodulation that shapes neuronal circuitry in response to sensory input. The objective of this review is to highlight current research investigating the mechanisms of primary cilium-regulated metabolic drives for maintaining energy homeostasis.

## Introduction

Obesity and overnutrition are common metabolic challenges of the 21st century. Obesity induces blood-brain barrier damage that is associated with lipid peroxidation and inflammation (Salameh et al., 2019). With time, this leads to neuronal dysfunction and behavioral phenotypes like anxiety and memory dysfunction that are deleterious to healthy cognitive aging (Takechi et al., 2017). Identifying mechanisms of metabolic-induced neuropathology are critical to our increasing aged and obese population.

Obesity is multifactorial: it can develop from an imbalance of social and environmental factors such as exercise or palatable diets and is influenced by the genetic landscape in which it occurs. Common obesity is polygenic, but in rarer circumstances can be associated with a single gene. Among efforts to elucidate the complete story of obesity pathology, the central nervous system has become a crucial investigative focus. The brain is the master regulator of energy homeostasis as it coordinates responses to humoral, metabolic, and neural signals from the periphery (Lee and Mattson, 2014). The hypothalamus is a particularly important integration hub that was first implicated in energy regulation by obesity-inducing lesions in the ventromedial nucleus (Brobeck, 1946). Neuronal response to metabolic cues is modulated by signaling at the primary cilium.

When ciliary assembly, maintenance, or function are compromised, clinically relevant ciliopathies develop at a combined frequency of 1:1000 live births (Zaghloul and Katsanis, 2009). Importantly, ciliopathies exhibit an extensive and overlapping spectrum of phenotypes—such as obesity—which are common in the general population. For example, between 72% and 86% of Bardet-Biedl Syndrome patients and 100% of Alström Syndrome patients develop early-onset obesity (Marshall et al., 2011; Forsythe and Beales, 2013). The role of ciliary dysfunction in obesity is supported by findings that the global ablation of primary cilia in rodent models induces adiposity characterized by excessive eating (hyperphagia) and hyperleptinemia (Davenport et al., 2007).

The primary cilium acts as an antenna amplifying signals *via* G-protein coupled receptor (GPCR) activity and high concentrations of intraciliary calcium that shift membrane polarization, allowing the neuron to integrate local environmental metabolic cues to inform downstream activity. As part of its signaling function the primary cilium uses unique trafficking intraflagellar transport machinery to insert/remove proteins into/from the ciliary membrane as trafficked proteins must contend with steric hindrance at the ciliary base due to fence-like scaffolding at the periciliary diffusion barrier (Berbari et al., 2008; Schou et al., 2015). The presence or absence of specific GPCRs at the ciliary membrane, i.e., inserted into the plasma membrane instead of the ciliary membrane, affects the cellular phenotype (Cook et al., 2021). Because dopamine and serotonin receptors are enriched in the primary cilium, this organelle is not only a hub for metabolic signaling, but also for catecholamine neuromodulation linking reward and motivation with metabolic requirements (Atkinson et al., 2015; Schou et al., 2015; Sheu et al., 2022). The objective of this review is to highlight current research investigating the role of the neuronal primary cilium in integrating metabolic function with behavioral drives.

## Primary cilium

Neuronal primary cilia are non-motile, ∼0.2–0.5 µm wide, 2–12 μm long organelles that project from the soma. A primary cilium is composed of a microtubule-based axoneme that emanates out of a basal body anchored *via* transition fibers. The basal body is composed of several Bardet–Biedl syndrome (BBS) proteins that make up the BBSome. The BBSome is tethered to the actin cytoskeleton and is critical for intraflagellar transport (IFT)-regulated trafficking of ciliary cargo (Berbari et al., 2008; Hernandez-Hernandez et al., 2013; Williams et al., 2014; Smith et al., 2020). Although the ciliary membrane appears to be continuous with the plasma membrane, lateral movement of membrane proteins is restricted (Vieira et al., 2006). Proteins destined for the ciliary membrane must dock at adapters near the ciliary base to pass through the ciliary transition zone, a diffusion barrier that provides steric hurdles to non-ciliary proteins (Nachury et al., 2010). Finally, the distinct lipid composition of the primary cilium alters its biophysical properties that determine signaling and protein trafficking (Nachury and Mick, 2019). The ciliary membrane is enriched with GPCRs embedded within lipid rafts next to calcium channels, making this organelle a highly dynamic sensor for the extracellular environment.

### Length and resorption

Primary cilium length can vary in response to homeostatic states or nutrient availability. In rat neurons, overexpression of serotonin receptor 5-HT6, a GPCR that localizes to the primary cilium, increases ciliary length, and this is associated in reduced dendritic arborization (Guadiana et al., 2013). Neuropeptide signaling at the primary cilium is classically represented by the GPCR for melanin-concentrating hormone (MCH), an orexigenic neuropeptide that links sleep, metabolism, and reward. Application of MCH decreases MCHR1+ primary cilia length which was blocked with application of G_i/o_ inhibitors (Kobayashi et al., 2021). Length changes and resorption occur in primary cilia in response to cell cycle transition. Cilia shrink during G1 to S phase until almost resorption during mitosis. Cell culture experiments demonstrate that nutrient state regulates length and ciliary resorption can be induced by nutrient availability (Lim et al., 2015). Resorption occurs with microtubule deacetylation that destabilizes the axoneme in addition to actin cytoskeletal destabilization (Kim et al., 2015; Lim et al., 2015; Ran et al., 2015). In some contexts, disassembly occurs in response to decapitation in which actin-dependent processes pinch off the tip of the primary cilium yielding a ciliary vesicle (Phua et al., 2017). However, because neurons are post-mitotic, their primary cilia have not been shown to undergo resorption.

Ciliogenesis occurs when membrane- bound vesicles emerging from the Golgi are recruited to the mother centriole where they fuse with proteins on the plasma membrane. The mother centriole is converted into the basal body, and proteins are recruited to establish the transition zone. Microtubules form at the basal body to become the growing axoneme, and cilia protein assembly is dependent on IFT and kinesin/dynein activity (Conduit and Vanhaesebroeck, 2020).

### Ciliary membrane

**FIGURE 1:**
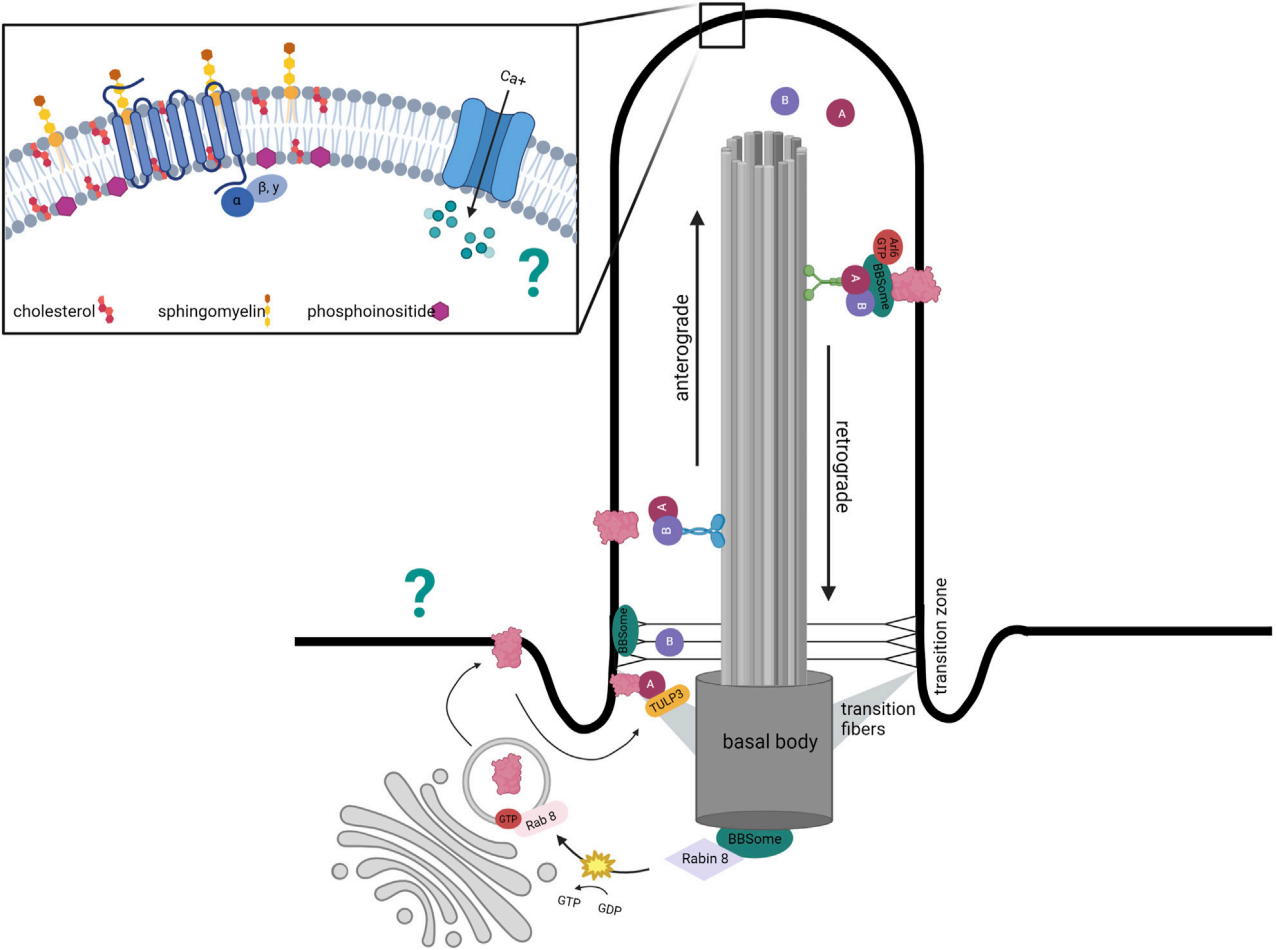
Distinct mechanism of ciliary protein transport. Structure of primary cilium to highlight distinct ciliary membrane composition in inset. Ciliary trafficking of receptors such as GPCRs involves vesicle budding from the Golgi that docks at the base of the cilium with intraflagellar (IFT)- mediated transportation *via* anterograde kinesin motors. IFT-A-mediated entry requires TUB like protein 3 (TULP3), a member of the Tubby family of proteins that interacts with the IFT-A core as an effector. The BBSome localizes to the basal body and transition zone and represents a docking point for proteins destined to enter the cilium. The BBSome has also been implicated in the exit of ciliary GPCRs out of the cilium as disruption of its functions leads to accumulation of GPCRs within the cilium. In addition to the BBSome, removal of proteins is mediated by retrograde transport with IFT complexes coupled to dynein motors. The question mark represents the uncertainty regarding how GPCR- associated cAMP and calcium oscillations are transmitted from the cilium to the rest of the neuron.

The ciliary membrane is distinct from the rest of the plasma membrane in its lipid composition. The relative proportions and spatial distribution of sphingolipids, phosphoinositides, and cholesterol in the ciliary membrane are essential for proper primary cilium function. Many GPCRs are embedded within lipid rafts, microdomains enriched with cholesterol and sphingolipids (Figure 1).

Sphingolipids have a ceramide building block that is modified at the endoplasmic reticulum depending on the plasma membrane destination (Olsen and Færgeman, 2017). One of the resulting derivatives, sphingomyelin is abundant in the nervous system plasma membrane and vital for dendritic arborization and myelination. Sphingolipids are also heavily represented in the ciliary membrane (Kaiser et al., 2020). The sphingomyelin: cholesterol ratio is especially high in the ciliary membrane where sphingomyelin sequesters accessible cholesterol in a mechanism that regulates canonical ciliary GPCR signaling (Myers et al., 2017; Kinnebrew et al., 2019). When sphingolipid metabolism is disrupted by blocking ceramide synthesis, organization of microdomains is also disrupted due to alterations in protein-lipid interactions. This can result in changes to membrane polarization when microdomains critical for ion channel function are altered by interfering with sphingolipid metabolism (Olsen and Færgeman, 2017).

Microdomain composition is critical for associating with lipid-binding domains on receptors/channels, and lipid modification of proteins determines membrane association (Resh, 2013). For example, when myristoylation (Emmer et al., 2009; Tao et al., 2009), palmitoylation (Emmer et al., 2009), or SUMOylation (McIntyre et al., 2015) is blocked, ciliary proteins fail to localize to the ciliary membrane. Palmitoylated acetylated tubulin colocalizes with ceramide-rich regions in the ciliary membrane, and inhibition of this post-translational modification was sufficient to impair ciliogenesis (Tripathi et al., 2021). However, not all lipid modifications have this effect as geranylgeranylation (Williams et al., 2014) of GFP restricted its trafficking to the ciliary proximal region of olfactory sensory neurons rather than to the ciliary membrane. Mislocalization of ciliary proteins can have robust behavioral effects (Yamakawa et al., 2021). When a ciliary GPCR that regulates energy homeostasis, melanocortin-4 receptor (MC4R), is retained subcellularly due to a mutation in the ciliary localization signal domain, ligand-induced activation is hindered. Mice with this mutation are obese and are unable to appropriately integrate metabolic signals at the hypothalamus (Siljee et al., 2018).

Although not particularly abundant compared to other membrane lipids, phosphoinositides are phospholipids enriched in the ciliary membrane. Phosphatidylinositol 4-phosphate [PtdIns(4)P], PtdIns (4,5)P_2_, PtdIns (3,4,5)P_3_, and PtdIns (3,4)P_2_ are phosphoinositides that define subdomains in the cilia. This is in part through their varied lipid-binding domains that determine recruitment of different effector proteins (Conduit and Vanhaesebroeck, 2020). One such lipid-binding domain critical to ciliary lipid membrane maintenance is the Pleckstrin Homology (PH). These domains are also present in phosphatidylinositol-4-phosphate adaptor protein-2 (FAPP2), which binds PtdIns (4)P *via* PH domains at the Golgi for transport. In cells, FAPP2 depletion hindered the formation of a primary cilium and was associated with vesicle accumulation at the microtubule organizing center (Vieira et al., 2006).

PtdIns (4)P provides structure to the ciliary membrane, and PtdIns (4,5)P_2_ levels regulate ciliary length and stability (Garcia-Gonzalo et al., 2015). Phosphatidylinositol 4,5-bisposphate [PtdIns (4,5)P_2_] exists along a gradient within the primary cilium with levels comparable to the plasma membrane at the base, but undetectable at the tip (Stilling et al., 2022). The compartmentalization occurs due to the coordinated activity of Tctn1 and Inpp5e. Tctn1 is a transition zone protein critical for ciliary localization of phosphoinositide 5-phosphatase (Inpp5e) which converts PtdIns (4,5)P_2_ into PtdIns(4)P. *Inpp5e* −/− mouse embryonic fibroblasts have a robust reduction in PtdIns(4)P levels and PtdIns (4,5)P_2_ is distributed along the ciliary membrane rather than restricted to the ciliary base. This shift in membrane lipid composition was associated with dysfunctional trafficking of ciliary proteins, including IFT-A whose ciliary levels depends on PtdIns (4,5)P_2_, and IFT-A is critical for intraflagellar transport of GPCRs (Garcia-Gonzalo et al., 2015). In humans, loss of *Inpp5e* function is associated with Joubert Syndrome, a multisystem ciliopathy characterized by organ dysfunction and cognitive impairment due to ciliary transition zone dysfunction (Dyson et al., 2017). Unsurprisingly, *Tctn1* mutations also cause Joubert Syndrome (Garcia-Gonzalo et al., 2011). Membrane lipid constitution is therefore essential to GPCR composition and length of the primary cilium.

### GPCR trafficking

The axoneme is encased by a phospholipid membrane studded with multiple GPCRs that are preferentially enriched in cilia trafficked *via* IFT trains. IFT trains are composed of subcomplexes—IFT-A and IFT-B—and protein motors that together carry cargo along the axoneme microtubules. Trains a) assemble at the base and undergo anterograde transport driven by IFT-B and kinesin-2 movement towards the positive end, b) are then remodeled at the ciliary tip, and finally c) exit through retrograde transport driven by IFT-A and dynein-2 movement towards the negative end. Many of the IFT components are necessary for cilia assembly (Lechtreck, 2015). Mutations in kinesin family member 3A (Kif3a)—a subunit of kinesin-2—or Ift88/Tg737—a subunit of the IFT-B complex—are commonly employed to conditionally ablate cilia in the laboratory (Davenport et al., 2007).

Considering that primary cilia are signaling hubs, IFT is also important for functional maintenance by trafficking receptors and downstream effectors. Ciliary protein trafficking is currently suspected to be a combination of IFT and diffusion with differences likely based on protein and cell type (Ye et al., 2013). IFT-dependent vesicular trafficking involves packaging proteins into vesicles, docking at the base of the cilium, and IFT movement to the tip (Rosenbaum and Witman, 2002). In addition to its role in anterograde transport, IFT-A has also been implicated in the entry of ciliary GPCRs. IFT-A-mediated entry requires TUB like protein 3 (TULP3), a member of the Tubby family of proteins that interacts with the IFT-A core as an effector (Figure 1) (Mukhopadhyay et al., 2010). siRNA depletion of Tulp3 inhibits the localization of ciliary GPCRs such as somatostatin receptor 3 (SSTR3) and melanin concentrating hormone receptor 1 (MCHR1) (Mukhopadhyay et al., 2010). However, IFT-A and TULP3 are not the only mediators of ciliary protein trafficking. For example, a complex of ciliary localizing proteins called the BBSome is also involved in other aspects of the vesicular trafficking pathway as will be discussed.

Overall, elucidating the mechanisms by which receptors and their effectors are trafficked to primary cilia is central to delineating how ciliary structure contributes to cilia-regulated energy homeostasis. For example, tubby mice—which exhibit a loss-of-function mutation in the *TUB* gene, the founding member of the Tubby family—manifest an obese phenotype (Coleman and Eicher, 1990), and homozygous mutation in *TUB* has been associated with early-onset obesity in humans (Borman et al., 2014).

### The BBSome

The BBSome complex is named for Bardet-Biedl Syndrome (BBS), a pleiotropic, autosomal recessive ciliopathy. BBS is characterized by retinal degeneration, renal dysfunction, and early-onset obesity, but also presents with hypogonadism, polydactyly, and learning difficulties (Forsythe and Beales, 2013). Renal dysfunction and obesity are the primary clinical concerns. Newborns typically exhibit normal birth weights; however, diffuse adiposity rapidly ensues by early childhood and adult patients exhibit truncal obesity (Forsythe and Beales, 2013; Pomeroy et al., 2021). Heterozygous carriers are also predisposed to obesity (Croft et al., 1995).

BBS genes either encode the subunits of the BBSome or are involved in its formation and activation. The BBSome is a heterooctameric protein complex implicated in ciliary structure and function (Loktev et al., 2008). The core complex is composed of subunits BBS1/2/4/5/7/8/9/18 and is assembled by a separate chaperonin-like complex containing BBS6/10/12 (Nachury et al., 2007; Loktev et al., 2008; Seo et al., 2010). During formation, BBS7 is stabilized by the chaperonin-complex so that it can interact with BBS2. The remaining core forms through protein-protein interactions. BBS1 and BBS4 are the most peripheral core subunits, suggesting that they may mediate any functional BBSome interactions (Zhang et al., 2012). Due to this assembly process, the entire BBSome can become compromised by the absence of nearly any of the core subunits or chaperonin-complex components.

Disruption of BBS2 is specifically associated with adult obesity whereas BBS4 and BBS6 have been linked to both adult and childhood obesity (Benzinou et al., 2006). However, BBS1 and its prevalent missense mutation (M390R) are not associated with adiposity (Fan et al., 2004; Benzinou et al., 2006). Mouse models both support and conflict with these associations. At around 4 months, Bbs1M390R knock-in, *Bbs2* −/−, *Bbs4* −/−, *Bbs6* −/−, and *Bbs7* −/− mice developed obesity characterized by hyperphagia (Nishimura et al., 2004; Eichers et al., 2006; Davis et al., 2007; Zhang et al., 2013). Similarly, human BBS patients exhibit hyperphagic behaviors (Sherafat-Kazemzadeh et al., 2013). However, disruption of BBS3/ARL6—an activator of the BBSome complex—induced limited weight gain without hyperphagia (Zhang et al., 2011). *Bbs2* −/− and *Bbs*4 −/− mice displayed decreased energy expenditure (Rahmouni et al., 2008). Currently, it is unclear if altered food intake or energy expenditure are causes or consequences of obesity in BBS. For example, *Bbs2* −/− and *Bbs*4 −/− mice still gained significant weight when hyperphagia was eliminated by pair-feeding, suggesting that decreased energy expenditure may underlie the phenotype (Rahmouni et al., 2008). However, a cohort of human BBS patients exhibited no significant differences in energy metabolism when compared to other obese individuals (Grace et al., 2003).

In the search for the underlying mechanisms of the BBS obesity phenotype, a parsimonious role for the BBSome has not yet been identified. First, the BBSome is not required for ciliogenesis, revealing a dichotomy in which IFT mutations hinder cilia formation whereas BBS mutations do not (Mykytyn et al., 2004; Nishimura et al., 2004; Davis et al., 2007; Zhang et al., 2013). Importantly, the BBSome localizes to the basal body and transition zone (Blacque et al., 2004; Nachury et al., 2007; Williams et al., 2014). The BBSome supports ciliary signaling through trafficking ciliary proteins.

The BBSome regulates multiple steps of the ciliary vesicular trafficking process. It has been implicated in regulating the enrichment of GPCRs within the cilium. For example, SSTR3, NPY2R and MCHR1 fail to accumulate in neuronal cilia in multiple brain regions in the absence of BBS1, BBS2, and BBS4 (Berbari et al., 2008; Loktev et al., 2013), and BBSome disruption induces ciliary accumulation of DR1R, GRP19, and hedgehog pathway receptors (Domire et al., 2011; Ye et al., 2018; Stubbs et al., 2023). Overall, it appears that the BBSome may select proteins for IFT-dependent trafficking and contribute to IFT train formation at the base and tip, potentially through stabilizing interactions between IFT-A and IFT-B (Blacque et al., 2004; Wei et al., 2012; Williams et al., 2014). The process of GPCR enrichment in neuronal primary cilia also relies on TUB and TULP3 proteins (Sun et al., 2012; Loktev et al., 2013). Similarly to BBS mutations, mice with TUB or TULP3 also develop obese phenotypes (Sun et al., 2012). A synthesized model of ciliary trafficking suggests that a) BBS1 in association with Rabin8—a guanine nucleotide exchange factor—activates Rab8 to promote vesicle docking at the ciliary base followed by b) direct protein entry into the cilia *via* IFT-A/TULP3 and finally c) the formation of a BBSome complex mediates the retrieval and export of specific proteins through the transition zone (Jin et al., 2010; Ye et al., 2018; Nachury, 2018). Thus, the underpinnings of the BBS obesity phenotype likely stem from the mislocalization and impaired signaling of ciliary membrane proteins whose trafficking is BBSome-dependent.

An attractive mechanism is the impaired localization of LepRb. First, hyperleptinemia and leptin resistance are present in many BBS mouse models such as *Bbs2* −/−, *Bbs4* −/−, and *Bbs6* −/− (Rahmouni et al., 2008). In humans, BBS patients exhibit leptin levels that are greater than what would be expected for their degree of adiposity (Feuillan et al., 2011; Büscher et al., 2012). Moreover, the hyperleptinemia appears causative considering that leptin levels were increased both before the onset of obesity and after weight normalization by calorie restriction (Rahmouni et al., 2008; Seo et al., 2010). Experiments have further demonstrated that resistance was not due to the inability of leptin to cross the blood-brain barrier but could rather be attributed to blunted STAT3 phosphorylation (Seo et al., 2009). BBS1 interacts with the C-terminal domain of LepRb—and the M390R mutation greatly impairs this interaction (Seo et al., 2009). Although BBS1 is the point of interaction, the entire BBSome was recently found to be necessary for trafficking LepRb not to the cilia as had been predicted, but rather to the periciliary plasma membrane (Guo et al., 2016). The mislocalization of LepRb underlies leptin resistance and is sufficient to induce obesity as demonstrated by significant weight gain in mice lacking BBS1 in neurons expressing LepRb (Guo et al., 2016). Thus, these studies have illuminated a role for the BBSome in trafficking proteins not only into cilia, but also to periciliary regions.

### Second messengers: Calcium and cAMP

The primary cilium structure is ideal for maintaining differential calcium gradients. The cytoplasmic volume is about 30,000x that of the primary cilium, and intraciliary calcium concentration is about 6X the cytoplasmic concentration ([Ca]_cilia_ ∼ 600 nM and [Ca]_cytosol_ ∼ 100 nM). The ciliary membrane is highly enriched in calcium channels like polycystin (PC)- and transient receptor potential cation (TRP)- family members (Decaen et al., 2013; Delling et al., 2013; Saternos et al., 2020). The small volume coupled with the density of calcium-permeant channels allows 200–300 calcium ions to generate a molarity 1 µM [Ca]_cilia_ making this an ideal environment for amplifying Gα_q_ – coupled receptors that when activated generate diacylglycerol and Ins (1,4,5)P_3_ in addition to the opening of TRP channels (Ritter and Hall, 2009).

Not only do intraciliary calcium levels regulate adenylyl cyclase 3 (ADYC3) signaling, but ADCY3 regulates intraciliary calcium activity. ADCY3 is a downstream effector of GPCR activation concentrated in primary cilia of olfactory neurons and critical to metabolism and contains a calcium-binding domain (Moore et al., 2018). This ADCY isoform is also found in osteoclast primary cilia where it regulates to fluid shear stress-induced calcium activity during bone growth (Halls and Cooper, 2011; Moore et al., 2018). Calcium bound- calmodulin and calcium alone have been shown to inhibit ADCY3 activity (Sadana and Dessauer, 2009).

ADCY3 levels in primary cilia control intraciliary cyclic adenosine monophosphate (cAMP) levels—whether or not intraciliary cAMP is distinct from cytosolic cAMP is unclear as reports have been conflicting (Moore et al., 2016; Jiang et al., 2019; Sherpa et al., 2019). Sequestration of these second messengers facilitates temporal and spatial signal integration. Transmission of both cAMP and calcium waves from the primary cilium signaling into the cytosol regulate neuronal excitability although the mechanism by which this occurs remains to be clarified (Green and Mykytyn, 2010). It is tempting to speculate that these signals are transmitted *via* inward rectifying potassium channels as G-protein coupled receptor inward rectifying potassium channels (GIRKs) are in close proximity to GPCRs and allows an efflux of potassium that hyperpolarizes the neuron (Lüscher and Slesinger, 2010). Similarly, PC channels in the primary cilium can be primed with high intraciliary calcium levels and are permeable to Na^+^ and K^+^ (Liu et al., 2018). An ideal location for such channels to propagate second messenger signals from the ciliary GPCR activity to the rest of the cell is at the base of the cilium, and potassium channels that regulate neuronal excitability have been found in the ciliary pocket and at the primary cilium base. Mutations to inward rectifying- and voltage-gated potassium channels are associated with impaired ciliogenesis (Sánchez et al., 2016; Napoli et al., 2022) indicating that sensitivity to local changes in voltage is a requirement for primary cilia.

Although not localized to the primary cilium *per se*, hypercholesteremia led to upregulation of membrane-bound cholesterol- associated GIRK activity in rat hippocampus that modified neuronal excitability. GIRK localization within cholesterol-enriched lipid rafts at membrane can be explained by their multiple cholesterol-binding motifs (Bukiya et al., 2017). Associated with G_q_ activity, PtdIns (4,5)P_2_ levels also regulate inward rectifying potassium channels. As previously mentioned, PtdIns (4,5)P_2_ levels exist along a gradient with the highest concentration at the ciliary base. Depletion of PtdIns (4,5)P_2_ from Gq – associated phospholipase C activity inhibits GIRKs in atrial myocytes, and supplementation with PtdIns (4,5)P_2_ reduced this inhibitory effect (Meyer et al., 2001).

The functional organization and relative size constraints of the primary cilium with its densely packed GPCRs in close apposition to calcium channels within cholesterol-rich lipid rafts allows for nuanced transmission to the neuronal soma. Ciliary activity must be integrated with inward-rectifying potassium channels and fluctuating PtdIns (4,5)P_2_ levels at the ciliary base to before spreading to the rest of the plasma membrane to mediate polarization. Primary cilium- induced hyper- or depolarization leads to downstream adjustments to intercellular circuitry.

## Interneuronal connectivity

In the brain, several neuromodulators have ciliary GPCRs, including the serotonergic receptor 5-HT6R; the dopaminergic receptors D1R, D2R, D5R; neuropeptidergic receptors for neuropeptide Y NPY2R, NPY5R and somatostatin receptors SSTR1-5 (Green and Mykytyn, 2010; Lukomska et al., 2020). Disruption of these signaling pathways *via* alterations to ciliary structure or function shifts neuronal connectivity. Dopaminergic tone is essential for short- and long-term maintenance of neuronal health as dopamine metabolites are oxidants (Meiser et al., 2013) and dopaminergic and GABA-ergic interneurons have high tonic activity. Therefore, ciliary localized receptors are poised to be integral to interneuron activity regulation.

This is elegantly depicted by selective deletion of Arl13b, a GTPase critical for ciliary signaling. Deletion of Arl13b disrupts signaling without affecting ciliary structure. Arl13b deletion from striatal parvalbumin+ and somatostatin+ interneurons was associated with altered morphology of inhibitory interneuronal networks that reduced perisomatic synaptic contact sites on medium spiny neurons (MSNs). Decreased perisomatic contacts from interneurons onto MSNs shifted the excitability/inhibitory tone with reduced miniature inhibitory postsynaptic currents without affecting miniature excitatory postsynaptic currents from sampled MSNs (Guo et al., 2017). Similarly, mutations in *Arl13b* and *Inpp5e* cause ciliopathies that fall under Joubert Syndrome related disorders and lead to aberrant axonal tracts in mouse models that mimics human axonal tract defects (Guo et al., 2019a). ARL13b is also dependent on TULP3 for its trafficking to the cilium and its mislocalization may underlie some of the neural defects in Tulp3 KO mice (Palicharla et al., 2023).

Disruption of BBSome signaling in Bbs4^−/−^ mice inhibits proper D1R trafficking causing accumulation of D1R in the primary cilium (Domire et al., 2011). Dysfunctional D1R at the primary cilium due to either conditional knock-out of primary cilia in D1R+ neurons or by eliminating *Bbs1* from D1R+ neurons in which loss of D1R from primary cilia or abnormal accumulation of D1R in the primary cilia lead to inappropriate striatal signaling. These mice are obese in part due to reduced locomotor activity in the absence of hyperphagia (Stubbs et al., 2022). Removing primary cilia from dorsal striatal neurons had several interesting behavioral outcomes including increased repetitive behaviors and deficits in sensorimotor gating (Alhassen et al., 2022a) indicating atypical striatal circuitry. However, not all neuronal cilia loss models cause locomotor changes and obesity phenotypes. Removal of cilia from GAD2-expressing GABAergic neurons resulted in no changes to basal locomotor activity, however mutant mice showed reduced body weight compared to wildtype littermates (Ramos et al., 2021).

## Primary cilia integrate peripheral signals at hypothalamic circuitry

The contribution of impaired ciliary signaling to obesity *via* disrupted energy expenditure suggests that primary cilia are important for hypothalamic responses to leptin. Leptin is an afferent signaling molecule that is released in proportion to fat stores to communicate a fed state, evoking a net anorexigenic response *via* short-term suppression of food intake and long-term stimulation of energy expenditure. It is secreted directly from adipose tissue, enters the bloodstream, and crosses the blood-brain barrier to bind to the LepRb splice form of leptin receptors which is primarily expressed in the hypothalamus (Gorska et al., 2010). Polymorphisms within LepRb have been associated with increased adiposity in multiple populations (Thompson et al., 1997; de Oliveira et al., 2013; Marginean et al., 2016), and mice heterozygous for loss-of-function mutations in the genes that encode for leptin and leptin receptors develop increased body fat (Chung et al., 1998).

The well-characterized action of leptin occurs within the leptin-melanocortin pathway (Baldini and Phelan, 2019). Here, leptin binds directly to the LepRb receptors of neurons in the arcuate nucleus (ARC). First, it activates proopiomelanocortin (POMC)-expressing neurons to stimulate the release of anorexigenic peptides such as alpha melanocyte stimulating hormone (α-MSH). Simultaneously, it inhibits agouti-related peptide (AgRP)-and neuropeptide Y (NPY)-expressing neurons to suppress the release of orexigenic peptides such as AgRP and NPY. AgRP, NPY, and α-MSH are released in the paraventricular nucleus (PVN) where α-MSH activates melanocortin-4 receptors (MC4R) and AgRP inhibits the constitutive activity of MC4R. When activated, LepRb can engage with several different pathways through the autophosphorylation of JAK2. The STAT3 pathway, initiated by phosphorylation of Tyr1138 on LepRb, promotes POMC expression to regulate food intake (Bates et al., 2003; Baldini and Phelan, 2019).

Leptin prepares the hypothalamus for its anorexigenic communication by modulating neuronal structure both in development and adulthood. Support for this comes from obese leptin-deficient (ob/ob) mice which exhibit a reduced density of ARC projections that can be rescued by leptin treatment during a postnatal period, but not in adulthood (Bouret et al., 2004). This suggests that leptin acts within a critical period to drive aspects of feeding circuitry formation. The disruption of ARC projections is paralleled in LepRb deficient (db/db) mice, indicating that LepRb mediates leptin’s developmental actions (Bouret et al., 2012). The modulation of ciliary length in adulthood is also a critical component of leptin’s anorexigenic signaling. For example, leptin treatment of N1 hypothalamic cells *in vitro* increased ciliary length up to 3 μm. One mechanism of leptin-induced cilia elongation is mediated through reduced density of F-actin fibers, which act as a blockade to both ciliary vesicular transport and membrane remodeling (Kang et al., 2015; Smith et al., 2020). Conversely, the length of cilia is reduced under conditions of low leptin or leptin insensitivity such as diet-induced obesity, ob/ob and db/db mice, or after 36 h of fasting (Han et al., 2014). Thus, longer cilia seem to be important for sufficient leptin signaling in a subset of hypothalamic neurons.

Logically, obesity might be expected to be correlated with low leptin levels; however, this is only the case for 10% of obese individuals (Ahima, 2008). Instead, hyperleptinemia develops into leptin resistance in an overwhelming number of obese patients and rodent models. Resistance is characterized by a lack of anorexigenic response despite high levels of circulating leptin and can either be primary or secondary to the development of obesity (Klok et al., 2007). Attractive mechanisms for causative resistance include defective leptin transport across the blood-brain barrier, underdeveloped hypothalamic circuitry, LepRb signaling cascade dysfunction or defects in downstream projections. Impairment of ciliary structure and signaling appears to contribute to leptin resistance in obesity pathology.

### Arcuate nucleus

**FIGURE 2:**
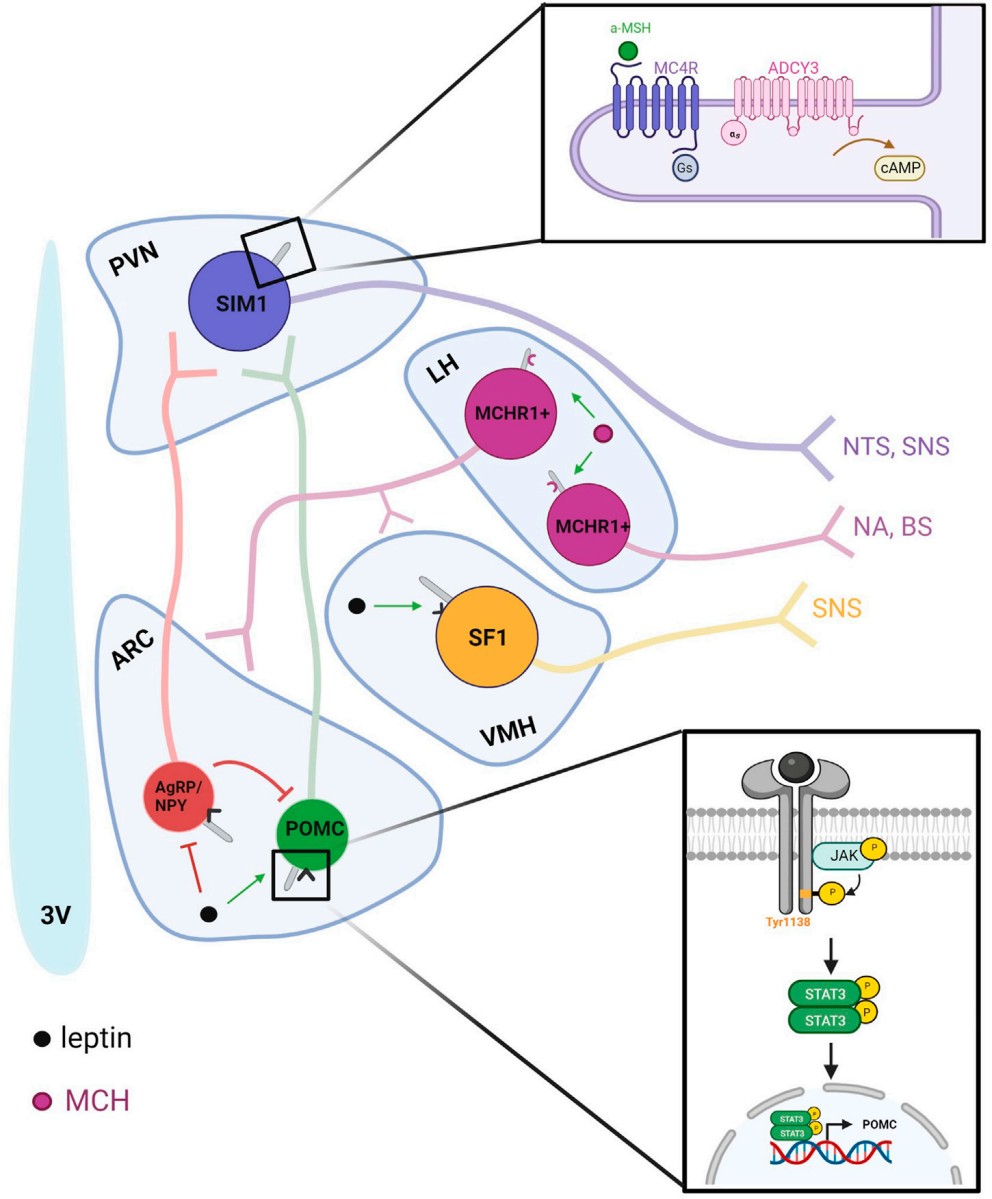
Ciliary-specific signaling in the hypothalamus integrates peripheral nutrient signals with neuronal output and connectivity. Leptin released from the periphery is detected by primary cilia in the hypothalamus. The leptin-melanocortin pathway begins in the ARC with the stimulation of POMC-expressing neurons and the inhibition of AgRP/NPY-expressing neurons. POMC-expressing neurons release α-MSH, which then binds to MC4R to increase satiety. Within the hypothalamus, MCH also regulates energy homeostasis as an orexigenic peptide that is upregulated during fasted states to increase food intake. In addition to the zona incerta, MCH is expressed in the lateral hypothalamus and sends projections locally to the ARC and VMH and to regions such as the brain stem and nucleus accumbens to modulate autonomic and hedonic aspects of food intake.

The leptin-melanocortin pathway begins in the arcuate nucleus (ARC) with the stimulation of POMC-expressing neurons and the inhibition of AgRP/NPY-expressing neurons (Figure 2). Signaling through POMC neurons is particularly necessary for leptin’s anorexigenic effects due to the production of α-MSH, an anorexigenic neuropeptide cleaved from the POMC precursor. The absence of leptin receptors from POMC neurons induces obesity associated with decreased POMC mRNA, and POMC deficiency in humans is characterized by early-onset obesity (Krude et al., 1998; Krude et al., 2003). Disruption of BBS1 in POMC or AgRP neurons was sufficient to induce adiposity through a decrease in POMC expression and induced pluripotent stem cell-derived hypothalamic arcuate-like neurons with the most common BBS1 and BBS10 mutations subjected to leptin failed to increase p-STAT3 (Guo et al., 2019b; Wang et al., 2021a).

Embryonic ciliogenesis is important for the leptin-driven formation of POMC-associated feeding circuits which are unwired at birth. Ciliogenesis was disrupted in rodent POMC neurons at embryonic day 10 by conditionally knocking-out *Ift88*, and the subsequent reduction in dendrite formation and arborization impaired circuit development leading to hyperphagic-associated obesity. Ciliogenesis could also be disrupted by postnatal exposure to a leptin antagonist, suggesting that the postnatal leptin surge may stimulate hypothalamic ciliogenesis as a necessity for its regulation of POMC circuit development (Lee et al., 2020).

Because inhibiting ciliogenesis reduces lysosomal protein degradation, impaired autophagy may underlie the obesity phenotype (Lee et al., 2020). When autophagy is disrupted in POMC neurons, axon growth is impaired, and adiposity subsequently develops (Coupé et al., 2012)– indicating a bidirectional relationship between ciliogenesis and autophagy regulation (Yamamoto and Mizushima, 2021). High-fat diets, specifically enriched with palmitic acid blunts autophagy by increasing levels of SQSTM1 and reducing the transformation of LC3I-LC3II in POMC neurons. This is associated with lysosomal swelling but decreased autophagosome-lysosomal fusion critical for cellular turnover of lipids (Hernández-Cáceres et al., 2020). Further work from this same group found that application of palmitic acid also led to insulin resistance through increased Free Fatty Acid Receptor 1 (FFAR1), and FFAR1 inhibition reversed the palmitic acid-induced insulin resistance in hypothalamic neurons (Hernández-Cáceres et al., 2019). Unlike cholesterol, palmitic acid can enter through the blood-brain barrier and accumulates in hypothalamic neurons leading to inflammation only in male mice. Interestingly, male mice with palmitic acid-induced hypothalamic inflammation also developed myocardial dysfunction (Morselli et al., 2014). Whether this was due to a shift in hypothalamic control of sympathetic tone was not elucidated, but high-fat diets have been shown to induce autonomic disturbances (Kim et al., 2021).

### Paraventricular nucleus

Neuropeptides from ARC neurons are released in the paraventricular nucleus (PVN) where α-MSH agonizes melanocortin 4 receptors (MC4R) *via* G_s_-coupled cascades to inhibit feeding (Marsh et al., 1999)**.** Heterozygous loss-of-function mutations in MC4R are the most common monogenic form of severe obesity in humans (Vaisse et al., 2000); *Mc4r* −/− mice develop obesity associated with hyperphagia and hyperleptinemia (Huszar et al., 1997). Obesity-inducing lesions within the PVN have implicated local neurons in autonomic regulation which may underlie the hypothalamic modulation of feeding behavior (Leibowitz et al., 1981; Sakaguchi et al., 1988).

MC4R is localized to the primary cilia of Single-minded 1 (Sim1) neurons (Siljee et al., 2018). Cilia are necessary for the anorexigenic action of MC4R as ablation of cilia in the PVN reduces their response to agonists (Wang et al., 2021b). MC4R signaling also requires cAMP production by adenylyl cyclases. Compounding evidence has specifically implicated Type 3 Adenylyl Cyclase (*Adyc3*) and its protein product ADCY3 in hypothalamic energy homeostasis. First, polymorphisms in *Adyc3* have been associated with obesity in multiple populations (Nordman et al., 2008; Wang et al., 2010; Grarup et al., 2018). A global knockout of *Adcy3* in mice induces obesity characterized by hyperphagia and decreased physical activity (Wang et al., 2009). These mice also exhibit hyperleptinemia and a blunted response to leptin prior to significant weight gain, suggesting that leptin resistance may underlie the obese phenotype. Conversely, a gain-of-function mutation (M279I) protects mice against diet-induced obesity (Pitman et al., 2014). ADCY3 is enriched to primary cilia throughout the brain and is commonly used as a nearly ubiquitous marker for neuronal primary cilia (Bishop et al., 2007). Recently, ADCY3 was found to colocalize with MC4R in Sim1+ neurons (Siljee et al., 2018). Specific inhibition of ciliary ADCY3 using GPR88, a G_i_-coupled receptor specific to cilia that was artificially constructed to be constitutively active, resulted in a blunted response to an MC4R agonist and subsequent weight gain associated with hyperphagia (Siljee et al., 2018). Thus, ADCY3 is suspected to be the downstream cAMP producer necessary for the MC4R signaling pathway that reduces food intake.

Currently, both MC4R and ADCY3 localization appear to be BBSome-independent (Berbari et al., 2008). For example, Tectonic1 (TCTN1), a transition zone complex, and SUMOylation have been implicated in ADCY3 trafficking in mouse embryonic fibroblasts and olfactory sensory neurons respectively (Garcia-Gonzalo et al., 2011; McIntyre et al., 2015). The role of SUMOylation in ADCY3 trafficking supports nucleoporins and processes analogous to nuclear import in ciliary entry (Kee and Verhey, 2013; Lynne Blasius et al., 2019). Considering that some *Adcy3* −/− mice display primary leptin resistance, it is tempting to propose that cAMP deficiency may underlie obesity by blunting anorexigenic downstream responses.

### Ventromedial hypothalamus

Although the melanocortin system is a primary focus for leptin’s anorexigenic action in the hypothalamus, obesity in POMC LepRb −/− mice is only a fraction of that observed after global LepRb depletion. The ventromedial nucleus (VMH) of the hypothalamus has long been implicated in energy homeostasis as evidenced by studies in which lesions induce obesity. This adiposity is associated with secondary hyperphagia as lesioned mice still gain significant weight compared to controls even when pair-fed. However, lesions do produce decreased sympathetic nervous activity in brown adipose tissue, suggesting that adiposity may develop out of decreased energy expenditure (vander Tuig et al., 1982). Steroidogenic factor-1 (SF-1)-expressing neurons have been identified as another type of neuron directly activated by leptin (Dhillon et al., 2006). Ablation of LepRb in SF-1 neurons in mice induced mild obesity which was not characterized by hyperphagia. Like POMC ablation from LepRb positive neurons, this phenotype produced only 20% of the weight gain resulting from global LepRb depletion (Dhillon et al., 2006). In another study, mice that similarly lacked LepRb in SF-1 neurons did not show significant weight gain on a standard diet but did exhibit increased susceptibility to diet-induced obesity (Bingham et al., 2008)**.** These studies support a role for first order SF-1 neurons in leptin’s regulation of energy homeostasis that is likely additive to POMC neurons.

Primary cilia may modulate the sympathetic tone that the VMH exerts upon brown adipose tissue as a mechanism for controlling energy expenditure. Ciliary ablation on SF-1 neurons in mice induced obesity associated with decreased energy expenditure but not hyperphagia (Sun et al., 2021). Interestingly, the BBSome is also implicated in sympathetic regulation. Knocking out *Bbs1* in SF-1 neurons produced a similar obesity phenotype characterized by decreased energy expenditure (Rouabhi et al., 2021). These hyperphagia-independent obesity models are distinct from the phenotypes that are produced by damage to other hypothalamic nuclei and neurons, suggesting that obesity induced by VMH dysfunction is characterized by a causative decrease in energy expenditure.

An *Adcy3* knockdown in the VMH using Cre-recombinase methods produced hyperphagia-associated obesity in mice on a standard diet (Cao et al., 2016). In a separate study, ciliary ADCY3 knockdown in the VMH using CRISPR-Cas9 did not induce significant weight gain on a standard diet but did result in increased susceptibility to a high-fat diet in mice (Yang et al., 2022). Altered autophagy due to a deficiency of ciliary ADCY3 may contribute to the development of obesity. GABA type A receptor-associated protein (GABARAP), a cAMP-regulated protein implicated in substrate recruitment to autophagosomes, was shown to interact with ADCY3 in a cilia-dependent manner. Furthermore, GABARAP knockout produced comparable susceptibility to diet-induced obesity (Yang et al., 2022). Considering that reduced autophagy hinders the development of POMC neurons in the ARC, it would be intriguing for future studies to explore if reduced ADCY3-dependent recruitment to autophagosomes has a structural impact on SF1 development and VMH sympathetic projections.

Finally, mutations in Rpgrip1l, a ciliary transition zone associated protein, are also associated with obese phenotypes. Mutations in Rpgrip1l underlie Joubert related disorders in humans, and lead to obese phenotypes in mice (Wolf et al., 2007; Stratigopoulos et al., 2014; Stratigopoulos et al., 2016). Hypomorphism of RPGRIPl1, leads to a reduction in ADCY3 positive cilia, increased food consumption and decreased sensitivity to leptin signaling (Stratigopoulos et al., 2016). The effects of Rpgrip1l mutation may be in part, neurodevelopmental in nature. Congenital targeting of Rpgrip1l in POMC neurons results in decreased neuronal number and obesity, while adult onset of hypomorphic mutations does not cause adiposity. Teasing apart changes in neurodevelopment and signaling in adult neurons will be important for understanding the role of ciliary signaling in controlling feeding behaviors (Cox and Powley, 1981).

### Lateral hypothalamus

MCH plays a role in energy homeostasis as an orexigenic peptide that is upregulated during fasted states to increase food intake (Qu et al., 1996). In addition to the zona incerta, MCH is expressed in the lateral hypothalamus and sends projections locally to the ARC and VMH and to regions such as the brain stem and nucleus accumbens to modulate autonomic and hedonic aspects of food intake (Figure 2) (Luthin, 2007). These neurons also project to several olfactory regions; the olfactory bulb (OB), anterior olfactory nucleus (AON), piriform cortex (PC), and olfactory tubercle (OT), where it is poised to modulate olfactory information that could influence feeding behaviors. Interestingly, MCH knockout mice show some defects in olfactory driven behaviors, such as reduced ability to locate a food source, and decreased maternal behaviors (Vaisse et al., 2000). MCH also promotes sleep, and MCH neurons exhibit increased activity during rapid eye movement (REM) sleep (Morselli et al., 2014; Kim et al., 2021). Optogenetically stimulating MCH neurons can prolong REM sleep, while their inhibition can shorten sleep cycles in rodents (Marsh et al., 1999; Morselli et al., 2014). MCH activity at the primary cilium offers another dimension for integration of peripheral input in hypothalamic neurons through the olfactory system. Peripherally, MCHR1 is found on adipocytes and the pancreas (Macneil et al., 2013), linking metabolic states in the brain with energy supplies in the form of fats and insulin. Localization of MCHR1 to the primary cilium is essential for its function and mislocalization to the plasma membrane yields an altered transcriptional profile in cell models (Cook et al., 2021).

MCH levels are elevated in diet-induced obese rats, and chronic infusion is sufficient to produce obesity in mice under either standard or high-fat diets (Gomori et al., 2003; Elliott et al., 2004). Conversely, *Mch* −/− and *Mchr1* −/− mice are both lean (Shimada et al., 1998; Marsh et al., 2002). Considering this, it seems that for MCHR1 mislocalization to contribute to obesity in BBS, the receptor must still be able to signal outside of the cilia and may even exhibit a gain of function to promote hyperphagia (Vaisse et al., 2017). MCH signaling at MCHR1 has been shown to modulate ciliary length. MCH shortens primary cilia in hTERT-RPE1 cells and *in vivo* work has replicated these results with optogenetic and chemogenetic stimulation of MCH neurons in CA1, striatum, prefrontal cortex, and nucleus accumbens (Hamamoto et al., 2016; Alhassen et al., 2022b). Ciliary length modulation by MCH opposes leptin’s anorexigenic signaling which elongates cilia. Cilia are shortened in obese ob/ob mice where perhaps orexigenic signaling experiences greater disinhibition due to the absence of leptin. This raises the question of how shortening cilia is beneficial for orexigenic signaling and how its inhibition may contribute to obesity.

### Median eminence

The median eminence (ME) is a circumventricular organ known for its blood-brain barrier (BBB) permeability and as such represents a critical node for peripheral-central metabolic communication. MCH neurons in the lateral hypothalamus that regulate feeding behavior project to the ME where they regulate blood-brain barrier permeability through their contacts with tanycytes and endothelial cells. Tanycytes are specialized ependymal/glial cells lining the ventricles that regulate cerebrospinal fluid flow. The vasculature of the ME is distinct from the rest of the brain and is organized into fenestrated capillary loops in contact with tanycytic processes. During fasted states, in which MCH levels are high, ME capillary fenestration is increased due to vascular endothelial growth factor-A (VEGF-A) release from MCH+ neurons. In mice, optogenetic activation of MCH+ neurons increased leptin sensitivity measured by reduced food intake as well as permeability of microvessel loops in the ME (Jiang et al., 2020). MCH+ neurons also project into the cerebroventricular space. Clever trapping experiments for immunosequestration of MCH in the third ventricle that reduced MCH levels in the cerebrospinal fluid also reduced food intake in rats. This work was critical in demonstrating activity of MCH outside of its receptor through volume transmission rather than synaptically (Noble et al., 2018).

## Discussion

Neuronal primary cilia regulate metabolic regulation *via* specific localization of trafficking proteins, receptors, and secondary messengers. Research has indicated that aberrant ciliary signaling through mutations that affect ciliary length, protein localization, and ciliary membrane composition have robust, global effects on energy homeostasis. This is particularly true when signaling of hypothalamic neuronal primary cilia are disrupted due to their bidirectional relationship with peripheral nutrient signals at circumventricular organs. Targeted inhibition of ciliary-specific receptors may therefore represent an attractive tool for mitigating neuropathology related to metabolic dysfunction.
